# 51 Wireless Electroceutical Dressing for the Treatment of Biofilm Infected Burn Wounds

**DOI:** 10.1093/jbcr/irac012.054

**Published:** 2022-03-23

**Authors:** Kristo Nuutila, Victoria Diaz, Kristin Anselmo, Shomita Steiner, Chandan K Sen, Anders H Carlsson, Rodney K Chan, Sashwati Roy

**Affiliations:** U.S. Army Institute of Surgical Research, San Antonio, Texas; US Army Institute of Surgical Research, San Antonio, Texas; US Army Institute of Surgical Research, San Antonio, Texas; Indiana University, Indianapolis, Indiana; University of Indiana, Indianapolis, Indiana; The Metis Foundation, San Antonio, Texas; US Army Institute of Surgical Research, San Antonio, Texas; University of Indiana, Indianapolis, Indiana

## Abstract

**Introduction:**

Burn injuries are common to all military conflicts. In combat, eradication and prevention of burn wound infection is complicated by high rates of soft tissue contamination and prolonged delays to definitive stateside care. Furthermore, in the battlefield setting the salvage rate for infected burned extremities is low. Therefore, a simple, easy, non-invasive and rapid method to protect the wound, while also inhibiting infection, would represent a significant advance in the treatment of combat burn wounds. The purpose of this clinical trial was to investigate the efficacy of an FDA approved disposable and easily portable, wireless electroceutical dressing (WED) in the treatment of burn wounds. The hypothesis was that a low electric field generated by the moisture-activated WED will reduce infection load, improve graft survival and take, enhance wound healing and restore skin barrier function of biofilm infected wounds.

**Methods:**

A phase I, prospective, randomized, controlled clinical trial was performed to evaluate the efficacy of the WED dressing as compared to the standard of care (SoC) dressing to prevent and disrupt biofilms. Subjects were screened from inpatient admissions for traumatic burns >300cm^2^ in size. In total 38 subjects were enrolled to the study. Subject burn wounds were divided into two parts and randomized to receive either the SoC dressing or the WED dressing. Dressings were changed on day 4, removed on day 7 and the burns were followed for 30 days. Small biopsies were collected on days 4 and 7 for histology, SEM examination of biofilm and for quantitative bacteriological analysis. In addition, non-invasive wound imaging techniques were utilized to study wound healing. Furthermore, Vancouver scar scale and patient observer scar assessment were used to evaluate quality of healing.

**Results:**

The results showed that at the time of dressing removal, non-grafted burns that were treated with the WED dressing presented statistically significantly less biofilm in comparison to the SoC treated burns (p < 0.05). The results also demonstrated that the WED dressing was more efficient at eradicating biofilm than the SoC dressing. At the time of the dressing removal, biofilm score [0-3] had decreased in 48% of the WED dressing treated burns in comparison to 28% in the SoC treated burns. In terms of wound healing and quality of healing no significant differences were observed between the WED and the SoC dressings.

**Conclusions:**

This trial demonstrated that the WED dressing was more efficient against biofilm infection than the SoC dressing. In addition, the study concluded that the WED dressing performed equally well as the SoC in terms of burn wound healing.

